# Cerebrospinal fluid seed aggregation assay and Alzheimer biomarkers in a Dementia with Lewy Body cohort

**DOI:** 10.1002/alz70856_107531

**Published:** 2026-01-09

**Authors:** Lawrence S. Honig, Min Suk Kang, Jennifer Lamoureux, Karen Marder

**Affiliations:** ^1^ Columbia University Irving Medical Center, New York, NY, USA; ^2^ Amprion, San Diego, CA, USA

## Abstract

**Background:**

Dementia with Lewy bodies (DLB) can be a challenging diagnosis clinically. Accurate Alzheimer's disease (AD) diagnosis has been improved by cerebrospinal fluid (CSF), molecular‐imaging, and recently, blood‐based, biomarkers. But only very recently has the advent of α‐synuclein seed‐aggregation‐assays (SAA), been available as a DLB diagnostic biomarker. DLB and AD pathologies co‐occur, so characterization is particularly important, with accompanying therapeutic implications. We report results of a cohort, enrolled in a longitudinal DLB study, the performance and stability of CSF SAA biomarker status, and its relation to demographics and AD biomarkers.

**Method:**

As part of the NINDS‐supported PDBP program, DLB patients were annually followed: thirty had CSF, and of those 13 had at least 2 separate CSF sampling. CSF samples were analyzed in blinded fashion by Amprion (San Diego, CA) using the SAAmplify™‐αSYN test, and at Columbia for AD biomarkers using the Quanterix HD‐X platform.

**Result:**

For the 30 clinically‐diagnosed DLB participants, 23 (77%) were SAA‐positive. Eight participants had two, and five participants three CSF samples each. Testing was stable over time: the 3 SAA‐negative participants remained negative, and 10 SAA‐positive remained positive. Comparison of 23 SAA‐positive and 7 SAA‐negative individuals revealed no significant differences in age (71.1±7.6 vs 73.8±6.3 yr), male sex (91% vs 100%), nonwhite ethnicity (8/23 vs 0/7), or APOE4 carrier status (8/23 vs 1/7). Likewise there were no significant difference in CSF, and plasma, biomarker levels for Aβ40, Aβ42, total‐tau, phosphotau181, NfL, or Aβ42/Aβ40, tau/Aβ42, or phosphotau181/Aβ42 ratios. Overall, about 15‐30% of both groups met AD‐positive criteria depending on which biomarker was used. One of 30 cases, which was CSF SAA‐negative, had an autopsy, and showed no DLB pathology, but only AD pathology, with substantia nigra neurofibrillary degeneration.

**Conclusion:**

Determination of DLB status using CSF SAA test in this clinical DLB cohort revealed high (77%) positivity. Repeated CSF evaluations revealed that SAA measurements, whether negative or positive, remained consistent over 1‐2 years. For the few individuals who were SAA negative, there was no evidence of increased Alzheimer pathology by CSF biomarkers, although the one SAA negative case that came to autopsy was also negative for Lewy body pathology.

